# The psycho-behavioral correlates of a drive to be toned among female university students

**DOI:** 10.1186/2050-2974-3-S1-P12

**Published:** 2015-11-23

**Authors:** Mark Suffolk, Elizabeth Blodgett Salafia, Maegan Jones

**Affiliations:** 1 North Dakota State University, Fargo, ND, USA

## 

Maladaptive psycho-behavioral concomitants of an increased drive for muscularity among men are well-documented (Pope et al., 1997). In recent years, media outlets aimed at women have promoted the idea that the ideal female physique is toned and strong, with sculptured abs. We suggest that the male drive to become muscular and the female drive to become toned are similar. However, less is known regarding the psycho-behavioral characteristics of women attempting to become toned. Our qualitative study examined women's beliefs and attitudes towards female muscularity. Twenty-two female university students ages 18 to 32 participated in focus groups addressing what it means for a woman to be toned and the physique enhancement strategies used by women today. Participants frequently indicated that being “in shape” is a feminine body ideal and being toned equates to empowerment and self-worth. Participants noted that weight-training and replacing meals with protein shakes or food supplements were typical physique enhancing strategies. We suggest that the drive to be toned can lead to women employing self-destructive physique enhancing strategies similar to those utilized by men who demonstrate an obsessive drive to become muscular. Therefore, researchers examining female body image concerns should be cognisant of muscle-enhancing attitudes and behaviors.

